# Predicting COVID-19 progression in hospitalized patients in Belgium from a multi-state model

**DOI:** 10.3389/fmed.2022.1027674

**Published:** 2022-11-23

**Authors:** Elly Mertens, Ben Serrien, Mathil Vandromme, José L. Peñalvo

**Affiliations:** ^1^Unit of Non-Communicable Diseases, Department of Public Health, Institute of Tropical Medicine Antwerp, Antwerp, Belgium; ^2^Department of Epidemiology and Public Health, Sciensano, Brussels, Belgium

**Keywords:** multistate modelling, risk prediction model, COVID-19, hospital data, Belgium

## Abstract

**Objectives:**

To adopt a multi-state risk prediction model for critical disease/mortality outcomes among hospitalised COVID-19 patients using nationwide COVID-19 hospital surveillance data in Belgium.

**Materials and methods:**

Information on 44,659 COVID-19 patients hospitalised between March 2020 and June 2021 with complete data on disease outcomes and candidate predictors was used to adopt a multi-state, multivariate Cox model to predict patients’ probability of recovery, critical [transfer to intensive care units (ICU)] or fatal outcomes during hospital stay.

**Results:**

Median length of hospital stay was 9 days (interquartile range: 5–14). After admission, approximately 82% of the COVID-19 patients were discharged alive, 15% of patients were admitted to ICU, and 15% died in the hospital. The main predictors of an increased probability for recovery were younger age, and to a lesser extent, a lower number of prevalent comorbidities. A patient’s transition to ICU or in-hospital death had in common the following predictors: high levels of c-reactive protein (CRP) and lactate dehydrogenase (LDH), reporting lower respiratory complaints and male sex. Additionally predictors for a transfer to ICU included middle-age, obesity and reporting loss of appetite and staying at a university hospital, while advanced age and a higher number of prevalent comorbidities for in-hospital death. After ICU, younger age and low levels of CRP and LDH were the main predictors for recovery, while in-hospital death was predicted by advanced age and concurrent comorbidities.

**Conclusion:**

As one of the very few, a multi-state model was adopted to identify key factors predicting COVID-19 progression to critical disease, and recovery or death.

## Introduction

As of July 4, 2022, a number of 4,294,880 confirmed cases of COVID-19 have been reported in Belgium, with 128,814 associated hospitalisations, and 31,977 deaths (1). This unprecedented burden on the health care system exposed the need for tools to facilitate informed clinical decision on patients’ management, and resource allocation for effective health systems, particularly during outbreaks of uncertain aetiology and heterogeneous prognosis. A robust risk prediction model of COVID-19 progression after hospitalisation, and the identification of predicting factors for patients’ outcomes would be key for not only delivering targeted treatments and strategies in high-risk patients, and hereby potentially increase survival while managing the pressure on the health care system, but also needed for informed decision on futile medical care.

Toward this need, more than 100 prognostic models for patients diagnosed with COVID-19 have been developed using a variety of data sources from the early phases of the pandemic. Most were discussed to carry a high risk of bias and lacking external validation, as summarised in the living systematic literature review of Wynants and co-workers (2). Standard logistic regression was a frequently used tool to predict COVID-19 severity, though likely to introduce important selection bias if patient data are not available until discharge; a plausible situation during a pandemic where data mainly cover active cases who are still in the hospital. More recent COVID-19 risk prediction models, however, accounted for some of the data complexity by considering time-to-event outcomes, hereby incorporating the time elapsing before an event is experienced or the observation is censored, either using Cox proportional hazard models (3–9) or in a competing risk framework (10–12). However, to the best of our knowledge, only a very few prediction scores have been built using time-to-event data in a multi-state setting that should fit better hospital progression, as documented by an increasing number of studies using a multi-state approach in the COVID-19 context (13–17).

A multi-state model framework allows for the simultaneous investigation of clinically competing outcomes (e.g., discharged alive versus in-hospital death), as well as an intermediate progression to a critical disease state [transfer to intensive care units (ICU)], while also accounting for censoring due to loss of follow up (18). The analysis of patients’ progression during hospitalisation is particularly challenged by the presence of competing events, where the occurrence of one event prevents the observation of another, and methods should account for this if interested in the probability of failing from an event (19). In addition, the probability of experiencing a certain event is likely to be influenced by patient’s risk factors, including time-dependent factors that are generally present with progression of disease. When it is of interest to model non-fatal intermediate events, the competing risk model can be easily extended to a multi-state model for the analysis of successive events (18). When analysing hospital data, especially in the context of COVID-19, it is important to acknowledge the presence of competing risks and intermediate events using adequate statistical models to provide robust findings (17).

The objective of the present study is to adopt a tool for multi-state risk prediction and identifying the key factors predicting COVID-19 progression to critical disease status, and subsequent recovery or death, using nationwide hospital surveillance data from COVID-19 in Belgium. This model aims to predict individual probabilities of experiencing an intermediate (ICU stay) and final events (recovery or death) after hospital admission, using survival methods in a multi-state setting.

## Materials and methods

### Data source

Nationwide hospital surveillance data on COVID-19 patients in Belgium are since the beginning of the pandemic routinely collected by Sciensano, the Belgian Institute of Public Health. The methodology of the Belgian surveillance system has been previously described in detail (20). Briefly, the clinical survey collects individual data of patients hospitalised in Belgium with confirmed COVID-19, as reported through a structured questionnaire at hospital admission and discharge (and a third questionnaire at ICU discharge if appropriate), and hereby achieving a national coverage at patient level of around 65%. Information reported at admission include patient demographics (such as age, sex, residence in a retirement home), type of the hospital at admission, date of hospital admission, symptoms at admission, the presence of chronic pre-existing comorbidities and risk factors, including current smoking. Information reported at discharge include laboratory values at admission, COVID-19 related treatment during hospital stay, measures on critical disease state (such as the need for transfer to ICU, invasive ventilation support and/or extracorporeal membrane oxygenation (ECMO), with only dates available for ICU transfer), development of complications [such as a bacterial and/or fungal superinfection, pneumonia, and/or acute respiratory distress syndrome (ARDS)], date of discharge, and health status at discharge. The ICU occupancy (percentage of recognized ICU beds occupied by confirmed- and suspected COVID-19 patients) is reported through a different surveillance system, the surge capacity survey (20).

This COVID-19 clinical surveillance was authorised by an independent administrative authority protecting privacy and personal data, and was approved by the ethical committee of Ghent University Hospital (BC-07507), which waived the informed consent because of the anonymous and retrospective data analyses, and because it was considered an extra burden on the hospitals during the pandemic. Ethical approval for present data analyses was obtained from the Institutional Review Board of the Institute of Tropical Medicine (ITM) after revision of the research protocol (num. 1488/21, 06/04/2021).

### Study population

A total of 55,737 adult patients with a community- or non-hospital-acquired SARS-CoV-2 infection confirmed by polymerase chain reaction (PCR) and/or suggestive imaging alterations on chest CT combined with typical clinical presentation at admission in Belgium from March, 2020 to June, 2021 were considered for inclusion in the present study. From this, excluded were patients with incomplete or implausible dates of admission, ICU transfer and/or discharge (15%), admitted to a psychiatric/categorical hospital (0.2%), and those with missing data on complete sections of the surveys: i.e., missing all symptoms (0.1%), all comorbidities (0.2%), and all laboratory values (6%), leaving 44,550 patients for the risk prediction model after multiple imputation (MI). Missing data among important potential candidate predictors were handled by 10-fold MI using the “mice” package in R (21).

Similar analyses were performed in a dataset excluding patients with missing data on any of the potential candidate predictors (66%), this complete-case analysis included 18,994 hospitalised COVID-19 patients in Belgium with complete data on necessary outcome variables for the prediction model (i.e., length of hospital stay, the time from admission to ICU for those admitted to ICU, and status at discharge) and potential candidate predictors.

### Patient predictor variables

Candidate predictor variables were identified on the basis of the presence of existing clinical vulnerability criteria, as identified by the WHO, previous literature and expert knowledge, and included the following variables measured at hospital admission: patients’ demographics [age (in quartiles), sex, residence in a retirement home], prevalent risk factors and comorbidities (current smoking, pregnancy, obesity, high blood pressure, diabetes, chronic renal disease, cardiovascular disease, chronic lung disease, chronic liver disease, malignant solid neoplasms, haematological cancers, immunosuppression, and including the number of comorbidities in four categories), symptoms at admission (fever, upper respiratory complaints, lower respiratory complaints, gastro-intestinal complaints, anosmia, loss appetite, and typical symptoms of viral infection), laboratory values at admission [c-reactive protein (CRP), lactate dehydrogenase (LDH), lymphocyte count; all in quartiles], as well as hospital characteristics [hospital type (in three categories) and ICU occupancy at admission and at ICU admission (in quartiles)].

### Patient outcome variables

Patients were considered to have recovered when their status at discharged was reported as “recovered” or “other” (with the latter representing recovery at home, a revalidation centre or a nursing home), to have in-hospital death when reported as “died,” and to be lost to follow-up when status at discharge was “unknown” or “transferred”. Hospital length of stay was calculated as the time in days starting from hospital admission until date of hospital discharge (either recovery, in-hospital death, or lost to follow-up). Only transfer to ICU was captured in the database as a time-defined critical state of COVID-19, with time to critical disease state calculated as time between hospital admission and date of transfer to ICU. If ICU transfer was on the same day as hospital admission, then we assumed half a day in hospital before a transfer to ICU.

In the multi-state model, transfer to ICU was modelled as a transient state, using time from admission to ICU, and recovery and in-hospital death as absorbing states either after hospital admission or *via* a transfer to ICU using hospital length of stay.

### The multi-state prediction model framework in the hospital setting

A multi-state model in the hospital setting describes the course of hospital stay from admission to discharge, including intermediate events of disease progression among the hospitalised patients. The present analysis is built upon the same set-up of the multi-state model as devised in our previous work (17), but we have extended the multi-state methodology for the deployment of a risk prediction model.

**FIGURE 1:**
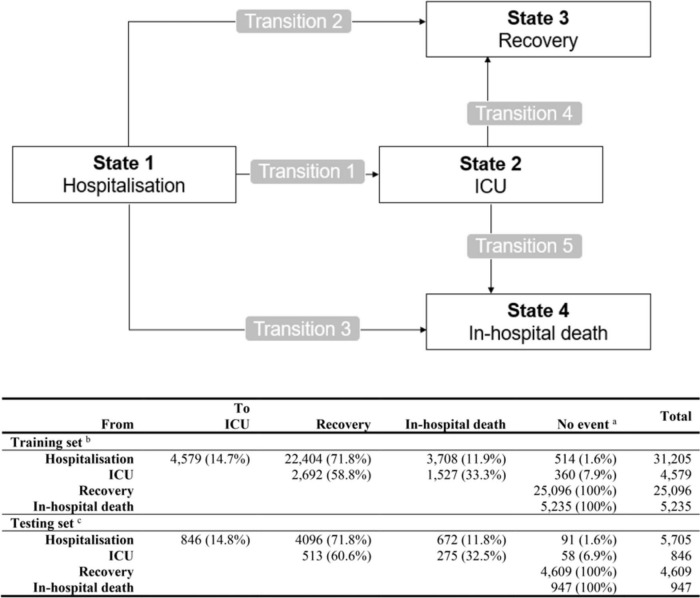
Schematic representation of the multi-state model with four states and five transition events, including the event matrix for training and Testing set. ^a^No event represents censored or absorbing state. ^b^Training set was taken in 10-fold for building the prediction model on stacked multiple-imputed data. ^c^Testing set for calculating prediction error includes complete cases only (excluding 57% of the Testing patients with missing data on any of the predicting variables).

In our multi-state model, patients entered in one initial state at day 0: State 1: “Hospitalisation.” From this State 1, they can either transition to a transient state: State 2: “ICU” (as a proxy for severe disease progression) or to one of the two absorbing states: discharged alive (State 3: “Recovery”) or in-hospital death (State 4: “Death”). From State 2, a transition to State 3 or State 4 was once again possible. Hereby, this four-state model incorporated five possible transitions: Transition 1: “Hospitalisation to ICU”, Transition 2: “Hospitalisation to recovery”, Transition 3: “Hospitalisation to death”, Transition 4: “ICU to recovery”, and Transition 5: “ICU to death”, as presented in Figure 1. In our model, being in the State 2 of ICU stay reflected a past transfer to ICU, but not necessarily a present stay at ICU, and also no back-transition to State 1 was considered.

### Training and testing set

The dataset is split 70/30 into a Training and Testing set. The prediction models were built in the Training set (including the variable selection from predefined candidate predictors and regression coefficient estimation). Predictions were made in the Testing set with the accuracy of the prediction evaluated using the Brier prediction error for multi-state models with pseudo-values to handle the presence of right censoring (22).

### Variable selection using the training set

Variable selection in the Training set was performed on the stacked MI data (23) using an elastic net procedure with alpha set to 0.5 and lambda within one standard error from the optimal lambda, determined by a 10-fold cross-validation using the “cv.glmnet” package in R (24).

### Prediction model adoption in the training set

This prediction model aims to predict individual state-occupation probabilities for the intermediate and the absorbing states at a future time t after hospital admission. To this end, we performed an elastic net Cox regression model for the variable selection of the five transition hazards, and subsequently, the four state-occupation probabilities were predicted by combining the (exponentiated) regression coefficients of the selected variables with the cumulative baseline hazards, as estimated by the Breslow estimator with the Aalen estimator of variance (25). Hereafter, we fitted also on the Training data a multi-state model without any covariates, i.e., a null model, with the aim to compare predicting performance of the prediction model with that of a null model.

### Prediction of state-occupation probabilities in testing set

The predictions of the state-occupation probabilities were calculated in R using the “mstate” package with “msfit” function to obtain the cumulative baseline hazards, and the “probtrans” function to compute the transition probabilities (25) in the Testing set for the model with the selected predictor variables and the null model.

### Risk prediction error measurement using testing set

To evaluate the prediction performance of the model, the prediction error based on the Brier Score was calculated as a measure of predictive accuracy that evaluates both discrimination and calibration simultaneously (26). Prediction error was calculated up to 30 days after hospital admission, since most of the patients had been discharged by then. Briefly, the Brier score is a function of the differences between the predictions and the observations and can be seen as the mean squared error of these differences. With the use of pseudo-values, the Brier score can also be estimated for survival models derived from incomplete data because of right censoring (27).

Pseudo-values were calculated in the Testing set, by directly modelling state-occupation probabilities in the Testing set by using a pseudo-value regression (28, 29). This approach replaces all observations, censored or not, with pseudo-values that are estimated by recalculating the Aalen-Johansen estimator derived from the entire Testing set and a Testing set with leave-one-out, repeatedly for each individual. This leave-one-out jack-knife method for the Aalen-Johansen estimator of the marginal state-occupation probabilities permits to assign pseudo-values for the state-occupation probabilities to each individual at each time point. In this way, a pseudo-value reflects the extent to which the overall marginal estimator is affected by the presence or absence of that individual in the set. This implies that pseudo-values are intuitively related to the covariates at the individual level, and thus contain information on how covariates of that individual affect the overall marginal estimator.

Subsequently, in the Testing set, the Brier prediction error estimated with pseudo-values for the state-occupation probabilities was calculated (22). The prediction errors were calculated for predictions made with the prediction models as well as for predictions made with the null model (i.e., the non-parametric multi-state model derived from the Training set) in order to quantify the relative reduction in the prediction error after covariate-inclusion.

### Building the nomogram

The selected predictor variables of the transition hazards were incorporated in a nomogram to predict the 2-, 3-, 7- and 14-day probability for the transitions from hospital admission to ICU-admission, to recovery and to in-hospital death. These days were chosen based on the distribution of time from hospital admission to ICU admission and total hospital length of stay, in particular day 2 and 3 mainly reflecting the need for an ICU transfer as 75% of the ICU patients are transferred before or at day 3 after hospitalisation, and day 7 and 14 mainly reflecting potential clinical outcomes of recovery or in-hospital death, in case of no transfer to ICU, as approximately one-third of the patients will have been recovered or died after 1 week of hospitalisation and up to 75% after 2 weeks. Using the “rms” package in R, the nomogram function provided for each variable a point score, ranging from 0 to 10 (low to high risk) and attributed according to the predictive importance of the selected variables on the specific transition probability, as represented by the format of the axes (30, 31).

## Results

### Descriptive characteristics of the study population

Patient’s characteristics were similar to those reported earlier in analyses using the Belgian COVID-19 surveillance data (17, 32–35) (Table 1). Approximately 85% of the hospitalised COVID-19 patients were aged ≥ 50 years and more than half of them were men. In general, the most frequent reported symptoms were lower respiratory complaints (69.7%), followed by fever (49.7%) and other symptoms typically associated with a viral infection (43.1%). A number of patients had a least one comorbidity with arterial hypertension (40.6%), cardiovascular disease (32.7%) and diabetes mellitus (23.8%) as the most common. Median length of hospital stay was 9 days. Approximately 15% of the patients were admitted to ICU with a median time from hospital to ICU admission of 1 day, and a total of 80.3% recovered, 16.9% died in the hospital and only 2.8% were lost to follow-up.

**TABLE 1:** Description of the information of COVID-19 patients according to health states during hospitalisation available from the Belgian COVID-19 hospital surveillance database between March 2020 and June 2021.

	Hospitalisation	Admission to ICU	Final health states		
			Recovery	In-hospital death	Lost to follow-up
	(*N* = 44,550)	(*N* = 6,520)	(*N* = 35,772)	(*N* = 7,536)	(*N* = 1,242)
**Age (years)**					
18–49	7,227 (16.2%)	939 (14.4%)	6,922 (19.4%)	107 (1.4%)	198 (15.9%)
50–69	14,460 (32.5%)	3,019 (46.3%)	12,745 (35.6%)	1,189 (15.8%)	526 (42.4%)
70–79	9,398 (21.1%)	1,797 (27.6%)	7,254 (20.3%)	1,843 (24.5%)	301 (24.2%)
80+	13,465 (30.2%)	765 (11.7%)	8,851 (24.7%)	4,397 (58.3%)	217 (17.5%)
Median (IQR)	70 (56, 82)	66 (56, 74)	67 (53, 79)]	82 (73, 87)	66 (56, 75)
Residence in retirement home	5,728 (12.9%)	301 (4.6%)	3,599 (10.1%)	2,077 (27.6%)	52 (4.2%)
Sex (males)	24,219 (54.4%)	4,375 (67.1%)	19,045 (53.2%)	4,412 (58.5%)	762 (61.4%)
Missing	129 (0.3%)	15 (0.2%)	105 (0.3%)	21 (0.3%)	3 (0.2%)
**Symptoms at admission**					
Lower respiratory	31,067 (69.7%)	5,419 (83.1%)	24,576 (68.7%)	5,594 (74.2%)	897 (72.2%)
Fever	22,131 (49.7%)	3,770 (57.8%)	17,833 (49.9%)	3,670 (48.7%)	628 (50.6%)
Typical viral infection	19,228 (43.2%)	2,912 (44.7%)	16,105 (45.0%)	2,637 (35.0%)	486 (39.1%)
Gastro-intestinal	11,538 (25.9%)	1,488 (22.8%)	9,966 (27.9%)	1,305 (17.3%)	267 (21.5%)
Anosmia	2,965 (6.7%)	435 (6.7%)	2,677 (7.5%)	195 (2.6%)	93 (7.5%)
Upper respiratory	3,150 (7.1%)	470 (7.2%)	2,740 (7.7%)	324 (4.3%)	86 (6.9%)
Loss of appetite	574 (1.3%)	238 (3.7%)	500 (1.4%)	69 (0.9%)	5 (0.4%)
**Pre-existing conditions**					
Cardiovascular disease	14,572 (32.7%)	2,058 (31.6%)	10,281 (28.7%)	3,915 (52.0%)	376 (30.3%)
Arterial hypertension	18,066 (40.6%)	2,890 (44.3%)	13,636 (38.1%)	3,913 (51.9%)	517 (41.6%)
Diabetes mellitus	10,609 (23.8%)	1,843 (28.3%)	8,020 (22.4%)	2,267 (30.1%)	322 (25.9%)
Chronic renal disease	5,981 (13.4%)	770 (11.8%)	4,109 (11.5%)	1,739 (23.1%)	133 (10.7%)
Chronic liver disease	1,139 (2.6%)	229 (3.5%)	856 (2.4%)	254 (3.4%)	29 (2.3%)
Chronic lung disease	6,853 (15.4%)	1,141 (17.5%)	5,084 (14.2%)	1,539 (20.4%)	230 (18.5%)
Neurological disorders	3,637 (8.2%)	361 (5.5%)	2,613 (7.3%)	950 (12.6%)	74 (6.0%)
Cognitive disorders	4,329 (9.7%)	214 (3.3%)	2,935 (8.2%)	1,320 (17.5%)	74 (6.0%)
Missing	2914 (6.5%)	549 (8.4%)	2,112 (5.9%)	729 (9.7%)	73 (5.9%)
Immunosuppressive condition	903 (2.0%)	214 (3.3%)	703 (2.0%)	177 (2.3%)	23 (1.9%)
**Cancer**					
Solid malignancies	4,193 (9.4%)	506 (7.8%)	3,014 (8.4%)	1,059 (14.1%)	120 (9.7%)
Haematological	870 (2.0%)	182 (2.8%)	582 (1.6%)	268 (3.6%)	20 (1.6%)
Transplant	199 (0.4%)	64 (1.0%)	157 (0.4%)	33 (0.4%)	9 (0.7%)
Missing	8,488 (19.1%)	1,222 (18.7%)	6,358 (17.8%)	1,939 (25.7%)	191 (15.4%)
Obesity	5,765 (12.9%)	1,430 (21.9%)	4,660 (13.0%)	860 (11.4%)	245 (19.7%)
Missing	2,914 (6.5%)	549 (8.4%)	2,112 (5.9%)	729 (9.7%)	73 (5.9%)
**Number of comorbidities**					
0	10,861 (24.4%)	1,343 (20.6%)	10,029 (28.0%)	555 (7.4%)	277 (22.3%)
1	11,231 (25.2%)	1,723 (26.4%)	9,359 (26.2%)	1,528 (20.3%)	344 (27.7%)
2	9,973 (22.4%)	1,548 (23.7%)	7,617 (21.3%)	2,080 (27.6%)	276 (22.2%)
3+	12,485 (28.0%)	1,906 (29.2%)	8,767 (24.5%)	3,373 (44.8%)	345 (27.8%)
**Risk factors**					
Pregnancy	479 (1.1%)	32 (0.5%)	465 (1.3%)	1 (0.0%)	13 (1.0%)
Current smoker	2,359 (5.3%)	371 (5.7%)	1,907 (5.3%)	359 (4.8%)	93 (7.5%)
Missing	17,491 (39.3%)	2,654 (40.7%)	13,885 (38.8%)	3,094 (41.1%)	512 (41.2%)
**Laboratory parameters at admission [Median (IQR)]**					
Lymphocytes (n/mmł)	800 (390, 1270)	720 (350.1136)	850 (440, 1320)	620 (260.1040)	610 (10.1080)
Missing	2,310 (5.2%)	449 (6.9%)	1,813 (5.1%)	431 (5.7%)	66 (5.3%)
LDH (IU/L)	334 (254, 457)	426 (318, 583)	321 (247, 432)	403 (296, 567)	377 (275, 534)
Missing	4,658 (10.5%)	585 (9.0%)	3,774 (10.6%)	743 (9.9%)	141 (11.4%)
CRP (mg/dL)	62 (25, 122)	198 (52, 180)	57 (21, 110)	96 (47, 166)	85 (33, 151)
Missing	390 (0.9%)	28 (0.4%)	358 (1.0%)	25 (0.3%)	7 (0.6%)
**Hospital characteristics**					
**Hospital type at admission**					
GH	31,069 (69.7%)	3,898 (59.8%)	24,856 (69.5%)	5,356 (71.1%)	857 (69.0%)
GHU	9,608 (21.6%)	1,547 (23.7%)	7,626 (21.3%)	1,662 (22.1%)	320 (25.8%)
UH	3,866 (8.7%)	1,073 (16.5%)	3,286 (9.2%)	515 (6.8%)	65 (5.2%)
Missing	7 (0.0%)	2 (0.0%)	4 (0.0%)	3 (0.0%)	0 (0%)
ICU occupancy [%, Median (IQR)]^a^	0.43 (0.25, 0.66)	0.41 (0.24, 0.62)	0.42 (0.25, 0.64)	0.46 (0.25, 0.68)	0.50 (0.26, 0.69)
Missing	309 (0.7%)	15 (0.2%)	247 (0.7%)	53 (0.7%)	9 (0.7%)
**Clinical features**					
Hospital length of stay [days, Median (IQR)]	9 (5, 15)	17 (10, 28)	9 (5, 14)	9 (5, 16)	8 (4, 16)
ICU transfer	6520 (14.6%)	6520 (100%)	3,816 (10.7%)	2,177 (28.9%)	527 (42.4%)
Time from hospital admission to ICU admission [days, Median (IQR)]	–	1 (0, 3)	–	–	
Final health status					
Discharged alive	35,772 (80.3%)	3,816 (58.5%)	35,772 (100%)	0 (0.0%)	0 (0.0%)
In-hospital death	7,536 (16.9%)	2,177 (33.4%)	0 (0.0%)	7,536 (100%)	0 (0.0%)
Lost to follow-up	1,242 (2.8%)	527 (8.1%)	0 (0.0%)	0 (0.0%)	1,242 (100%)

Values are numbers and percentage, or median and interquartile values. CRP, c-reactive protein; GH, general hospital; GHU, General Hospital with University characteristics; ICU, intensive care units; IQR, interquartile range; LDH, lactate dehydrogenase; UH, university hospital.

^a^ICU occupancy taken at admission for hospitalised patients without a transfer to ICU, and for ICU patients taken at the time of their ICU transfer instead.

Compared to non-ICU patients, the ICU-patients were slightly younger, and more frequently males, more likely to report symptoms of fever, lower respiratory complaints, and loss of appetite (but less gastro-intestinal complaints), to suffer from pre-existing conditions (particularly obesity, arterial hypertension, and diabetes mellitus), and to present lower levels of lymphocyte and higher levels of LDH and CRP. In addition, median length of hospital stay was more than double for those admitted to ICU (median 17 days in ICU-patients vs. 8 days in non-ICU-patients; data of non-ICU patients; not shown), and more patients experienced an in-hospital death (33.4 vs. 14.1%).

Compared with patients that recovered, the patients who died in the hospital were older, more frequently males, and more likely to report less symptoms (but more lower respiratory complaints), to suffer from pre-existing conditions, and to present lower levels of lymphocyte and higher levels of LDH and CRP. Median length of hospital say did not differ by final health states, but as compared with patients that recovered, the patients with an in-hospital death were more often transferred to ICU (28.9 vs. 10.6%), and also the patients that were lost-to-follow-up were more often transferred to ICU (42.4%).

### Risk prediction models in a multi-state setting

The estimates for the transition hazards for the risk prediction model of COVID-19 disease progression, as applied to 31,205 patients included in the training set, are presented in Table 2.

**TABLE 2:** Hazard ratios and 95% confidence intervals for transitions of COVID-19 disease progression in a multi-state risk prediction setting using the Training dataset with 10-fold multiple imputation.

Predictors	Transition 1	Transition 2	Transition 3	Transition 4	Transition 5
	Hospitalisation → ICU	Hospitalisation → Recovery	Hospitalisation → In-hospital death	ICU → Recovery	ICU → In-hospital death
**Age (years)**					
50–69 vs. < 50	1.20 (1.17; 1.24)	0.79 (0.78; 0.8)		0.63 (0.61; 0.65)	1.17 (1.09; 1.26)
70–79 vs. < 50	1.14 (1.10; 1.18)	0.51 (0.5; 0.52)	2.27 (2.18; 2.37)	0.45 (0.44; 0.47)	1.78 (1.65; 1.92)
80+ vs. < 50	0.43 (0.41; 0.45)	0.34 (0.34; 0.35)	4.50 (4.34; 4.68)	0.50 (0.47; 0.53)	3.08 (2.84; 3.33)
Sex (males)	1.30 (1.27; 1.33)	0.98 (0.97; 0.99)	1.23 (1.2; 1.26)	0.90 (0.88; 0.93)	
Residence in retirement home	0.63 (0.61; 0.66)	0.93 (0.91; 0.94)	1.86 (1.81; 1.90)		1.67 (1.57; 1.78)
**Symptoms at admission**					
Lower respiratory	1.38 (1.35; 1.42)		1.33 (1.30; 1.36)	0.95 (0.92; 0.98)	1.07 (1.03; 1.12)
Fever	1.04 (1.02; 1.06)	0.96 (0.95; 0.97)		0.96 (0.94; 0.99)	0.94 (0.90; 0.97)
Typical viral infection	0.90 (0.89; 0.92)	1.04 (1.03; 1.05)	0.93 (0.91; 0.96)		0.87 (0.84; 0.9)
Gastro-intestinal	0.81 (0.79; 0.83)	1.07 (1.06; 1.08)	0.81 (0.79; 0.84)	1.08 (1.05; 1.11)	0.97 (0.93; 1.01)
Anosmia	0.84 (0.81; 0.88)	1.17 (1.15; 1.19)	0.75 (0.70; 0.8)	1.04 (0.99; 1.09)	0.89 (0.83; 0.96)
Upper respiratory	0.90 (0.87; 0.94)	1.00 (0.99; 1.02)		1.02 (0.97; 1.06)	0.83 (0.77; 0.89)
Loss of appetite	1.74 (1.65; 1.85)		0.94 (0.82; 1.07)	1.55 (1.45; 1.65)	0.86 (0.76; 0.98)
**Pre-existing conditions**					
Cardiovascular disease	1.03 (1.00; 1.05)	0.95 (0.94; 0.96)	1.25 (1.22; 1.28)	0.99 (0.96; 1.03)	1.10 (1.06; 1.15)
Arterial hypertension	1.12 (1.10; 1.14)		1.00 (0.97; 1.02)	0.97 (0.94; 1.00)	0.90 (0.86; 0.94)
Diabetes mellitus	1.06 (1.04; 1.08)	0.97 (0.96; 0.98)	1.05 (1.02; 1.08)	0.95 (0.91; 0.98)	1.09 (1.04; 1.14)
Chronic renal disease		0.92 (0.91; 0.93)		0.95 (0.91; 1.00)	1.23 (1.17; 1.29)
Chronic liver disease	1.13 (1.07; 1.19)	0.90 (0.87; 0.92)	1.45 (1.36; 1.54)		1.19 (1.10; 1.29)
Chronic lung disease	1.01 (0.99; 1.04)	0.89 (0.88; 0.90)	1.01 (0.98; 1.04)	0.86 (0.82; 0.89)	1.27 (1.22; 1.33)
Neurological disorders	0.86 (0.82; 0.89)	0.83 (0.81; 0.84)	1.24 (1.2; 1.28)		
Cognitive disorders	0.68 (0.65; 0.71)	0.87 (0.86; 0.89)	1.15 (1.12; 1.18)		1.04 (0.96; 1.12)
Immunosuppressive condition		0.98 (0.95; 1.01)		0.9 (0.83; 0.97)	1.03 (0.94; 1.14)
**Cancer**					
Solid malignancies	0.88 (0.85; 0.91)	0.90 (0.89; 0.92)	1.27 (1.23; 1.31)	0.96 (0.91; 1.01)	1.03 (0.98; 1.10)
Haematological	1.18 (1.11; 1.25)	0.85 (0.82; 0.88)	1.32 (1.24; 1.4)	0.97 (0.89; 1.05)	1.52 (1.4; 1.66)
Transplant	1.23 (1.12; 1.35)			0.96 (0.85; 1.09)	1.27 (1.09; 1.48)
Obesity	1.46 (1.43; 1.49)			0.94 (0.91; 0.98)	0.92 (0.88; 0.96)
**Number of comorbidities**					
1 vs. 0	1.10 (1.08; 1.13)	0.92 (0.91; 0.93)	1.05 (1.00; 1.10)		1.13 (1.06; 1.20)
2 vs. 0	1.08 (1.05; 1.10)	0.85 (0.84; 0.87)	1.17 (1.11; 1.23)	0.90 (0.86; 0.94)	1.23 (1.14; 1.33)
3+ vs. 0		0.82 (0.80; 0.84)	1.12 (1.05; 1.19)	0.97 (0.91; 1.03)	1.40 (1.25; 1.57)
Pregnancy	0.91 (0.81; 1.03)	1.28 (1.23; 1.33)			0.77 (0.51; 1.16)
Current smoker		0.95 (0.94; 0.97)	1.00 (0.96; 1.04)	1.03 (0.98; 1.07)	1.06 (1.00; 1.12)
**Laboratory parameters at admission**					
**Lymphocytes (n/mmł)**					
Q2 vs. Q1		0.98 (0.97; 0.99)			
Q3 vs. Q1	0.89 (0.87; 0.91)	1.05 (1.03; 1.06)	0.83 (0.81; 0.85)	1.10 (1.07; 1.14)	0.89 (0.85; 0.92)
Q4 vs. Q1	0.88 (0.86; 0.90)	1.16 (1.14; 1.17)	0.79 (0.77; 0.82)	1.17 (1.13; 1.21)	0.98 (0.94; 1.03)
**LDH (IU/L)**					
Q2 vs. Q1			1.04 (1.01; 1.08)		
Q3 vs. Q1	1.31 (1.28; 1.34)	0.98 (0.97; 0.99)	1.19 (1.15; 1.23)	0.93 (0.90; 0.96)	
Q4 vs. Q1	2.02 (1.98; 2.07)	0.89 (0.88; 0.90)	1.81 (1.75; 1.86)	0.81 (0.78; 0.83)	1.20 (1.16; 1.25)
**CRP (mg/dL)**					
Q2 vs. Q1		0.92 (0.91; 0.94)	1.16 (1.12; 1.2)	0.96 (0.91; 1)	
Q3 vs. Q1	1.26 (1.23; 1.29)	0.90 (0.89; 0.91)	1.40 (1.35; 1.44)	0.87 (0.84; 0.91)	1.03 (0.98; 1.08)
Q4 vs. Q1	2.10 (2.05; 2.15)	0.82 (0.81; 0.83)	1.93 (1.87; 2.00)	0.76 (0.72; 0.79)	1.11 (1.07; 1.16)
**Hospital characteristics at admission**					
**Hospital type**					
GHU vs. GH	1.16 (1.14; 1.19)				1.16 (1.11; 1.20)
UH vs. GH	1.88 (1.83; 1.94)	1.04 (1.02; 1.05)	0.94 (0.9; 0.99)	1.09 (1.05; 1.13)	0.69 (0.65; 0.73)
**ICU occupancy**					
Q2 vs. Q1		1.01 (1.00; 1.02)			
Q3 vs. Q1	0.88 (0.86; 0.90)		1.14 (1.11; 1.17)		
Q4 vs. Q1	0.79 (0.77; 0.81)	0.97 (0.96; 0.98)	1.09 (1.06; 1.11)	0.92 (0.90; 0.95)	

CRP, c-reactive protein; GH, general hospital; GHU, General hospital University; ICU, intensive care units; LDH, lactate dehydrogenase; UH, university hospital; Q, quartile.

^a^ICU occupancy taken at admission for hospitalised patients without a transfer to ICU, and for ICU patients taken at the time of their ICU transfer instead.

Recovery after hospital admission (transition 2) was strongly positively predicted by pregnancy (HR: 1.28, 95% CI: 1.23–1.33), reporting symptoms of anosmia (1.17, 1.15–1.19), presenting high levels of lymphocytes (1.16, 1.14–1.17), while inversely by advanced age (50–69 years: 0.79, 0.78–0.80; 70–79 years: 0.51, 0.50–0.52; 80+ years: 0.34, 0.34–0.35), presenting two or more comorbidities (2: 0.85, 0.84–0.87; 3+ years: 0.82, 0.80–0.84), and in particular neurological disorders (0.83, 0.81–0.84), haematological malignancies (0.85, 0.82–0.88), cognitive disorders (0.87, 0.86–0.89), chronic lung disease (0.89, 0.88–0.90) and chronic renal disease (0.90, 0.87–0.92), as well as presenting high levels of CRP (0.82, 0.81–0.83) and of LDH (0.89, 0.88–0.90).

Strong predictors for an increased probability of ICU transfer (transition 1) were presenting high values for CRP (2.10, 2.05–2.15) and LDH (2.02, 1.98–2.07), being admitted to a University Hospital (1.88, 1.83–1.94), reporting symptoms of loss of appetite (1.74, 1.65–1.85), suffering from obesity (1.46, 1.43–1.49), reporting symptoms of lower respiratory complaints (1.38, 1.35–1.42) and being male (1.30, 1.27–1.33), while a lower risk was observed for aged 80+ years (0.43, 0.41–0.45), residence in a retirement home (0.63, 0.61–0.66), suffering from cognitive disorders (0.68, 0.65–0.71), and overloaded ICU occupancy (0.79, 0.77–0.81).

The probability of in-hospital death after hospital admission (transition 3) was mainly positively predicted by advanced age (70–79 years: 2.27, 2.18–2.37; 80+ years: 4.50, 4.34–4.68), a residence in retirement home (1.86, 1.81–1.90), presenting high levels of CRP (1.93, 1.87–2.00) and LDH (1.81, 1.75–1.86), reporting symptom of lower respiratory complaints (1.33, 1.30–1.36), presenting two or more comorbidities (2: 1.17, 1.11–1.23; 3+: 1.12, 1.05–1.08), and in particular chronic liver disease (1.45, 1.36–1.54), haematological (1.32, 1.24–1.40) and solid (1.27, 1.23–1.31) malignancies, cardiovascular disease (1.25, 1.22–1.28) and chronic neurological disorders (1.24, 1.20–1.26), while inversely by reporting symptoms of anosmia (0.75, 0.70–0.80) and of gastro-intestinal complaints (0.81, 0.79–0.84), and presenting high values of lymphocytes (0.79, 0.77–0.82).

For the transition to recovery after a transfer to ICU (transition 4), advanced age was identified as a strong inverse predictor (50–69 years: 0.76, 0.72–0.79; 70–79 years: 0.45, 0.44–0.47, 80+ years: 0.50, 0.47–0.53) as well as high levels of CRP (0.76, 0.72–0.79) and LDH (0.81, 0.78–0.83), while reporting loss of appetite (1.55, 1.45–1.65) as a positive predictor. For the transition to in-hospital death after a transfer to ICU (transition 5), the predictors for a higher probability were advanced age (70–79 years: 1.78, 1.65–1.92; 80+ years: 3.08, 2.84–3.33; residence in retirement home: 1.67, 1.57, 1.78) and suffering from comorbidities (2: 1.23, 1.14–1.33; 3+ year: 1.40, 1.25–1.57), and in particular haematological malignancies (1.52, 1.40–1.66), chronic lung disease (1.27, 1.09–1.48), transplant (1.27, 1.22, 1.33) and chronic renal disease (1.23, 1.17–1.29), while staying at a university hospital (0.69, 0.65–0.73) was the main predictor for a lower probability.

**FIGURE 2:**
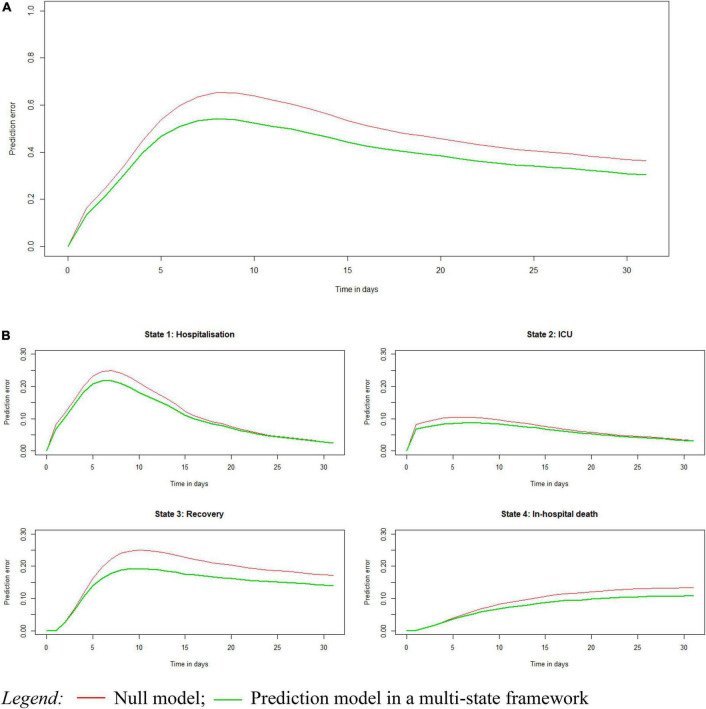
Prediction error of the multi-state prediction model as compared with a null model, calculated using the Brier score, overall **(A)** and stratified by state **(B)**.

Figure 2 shows the performance of the risk prediction model in the Testing set as compared to a null model. Overall, the prediction error for both the null and full model was increasing during the first days after hospitalisation, and started decreasing after 7 days to flatten after day 20. At 7 days after hospital admission, the prediction error was 0.64 for the null and 0.53 for the full model, pointing to a proportional reduction of 16% which was, on average, also observed for the following days. Stratified by health state, the prediction errors were taking a different course over time for each state, and followed the flow of patients during the hospital stay, i.e., a fast increase and a flattened decrease for the state of hospitalisation and ICU as well as for the state of recovery but at a slower speed, while only a flattened increase in prediction error for the state of in-hospital death.

### State probabilities of a reference patient versus a high-risk patient

**FIGURE 3:**
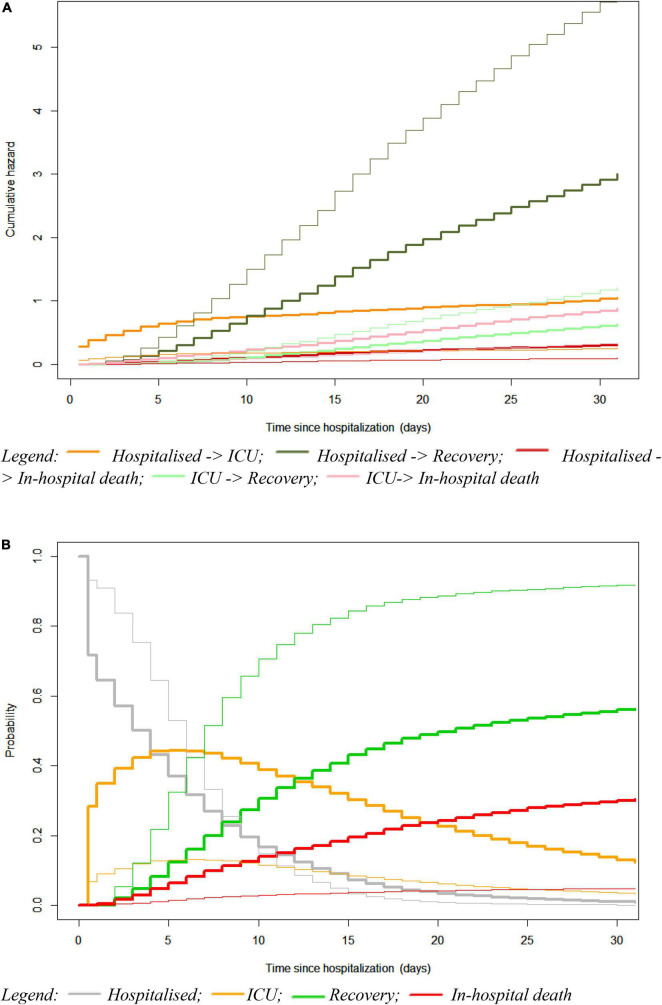
Plots for the cumulative transition hazards **(A)** and state-occupation probabilities **(B)** in a multi-state model considering a high-risk patient (tick solid line) versus a reference patient (thin solid line). The reference patient represents an average patient characterised by being male, aged between 55 and 69 years old, admitted to a general hospital with a medium-high ICU occupancy at hospital admission, experiencing lower respiratory complaints, having zero co-morbidities, being a non-smoker, and presenting high levels of lymphocyte and medium-high levels (Q3) of lactate dehydrogenase (LDH) and C-reactive protein (CRP). The high-risk patient represents an existing patient with the worst risk profile, while having the same age, sex, hospital type and ICU occupancy at admission as the reference patient.

Risk prediction outcomes were visualised using plots for the cumulative hazards for the five possible transitions and the final state-occupation probabilities, and in this case for a high-risk patient versus a reference patient (Figure 3). Overall, the cumulative hazards for recovery were markedly greater than that of in-hospital death as well as that of the transfer to ICU, which was associated with an increased hazard for in-hospital death and decreased hazard for recovery (Figure 3A). Transition hazards were, however, different between a high-risk and a reference patient. For a high-risk patient, a lower hazard to recovery was observed, independently of transfer to ICU, as well as a higher transition hazard for a transfer to ICU and in-hospital death after hospitalisation and after ICU. Similarly, patients with a high-risk profile had higher state-occupation probabilities for ICU, starting immediately after hospital admission and slowly levelling off at 5 days after hospital admission, and also had a higher probability for in-hospital death along with a lower probability for recovery (Figure 3B). However, for both patient profiles, the state-occupation probabilities of recovery were higher than those of in-hospital death, by which the recovery probability were showing an increase over time that stabilised after 15–20 days of hospitalisation and those of in-hospital death a gentle increase over the 30-day hospital stay.

### Nomogram

**FIGURE 4:**
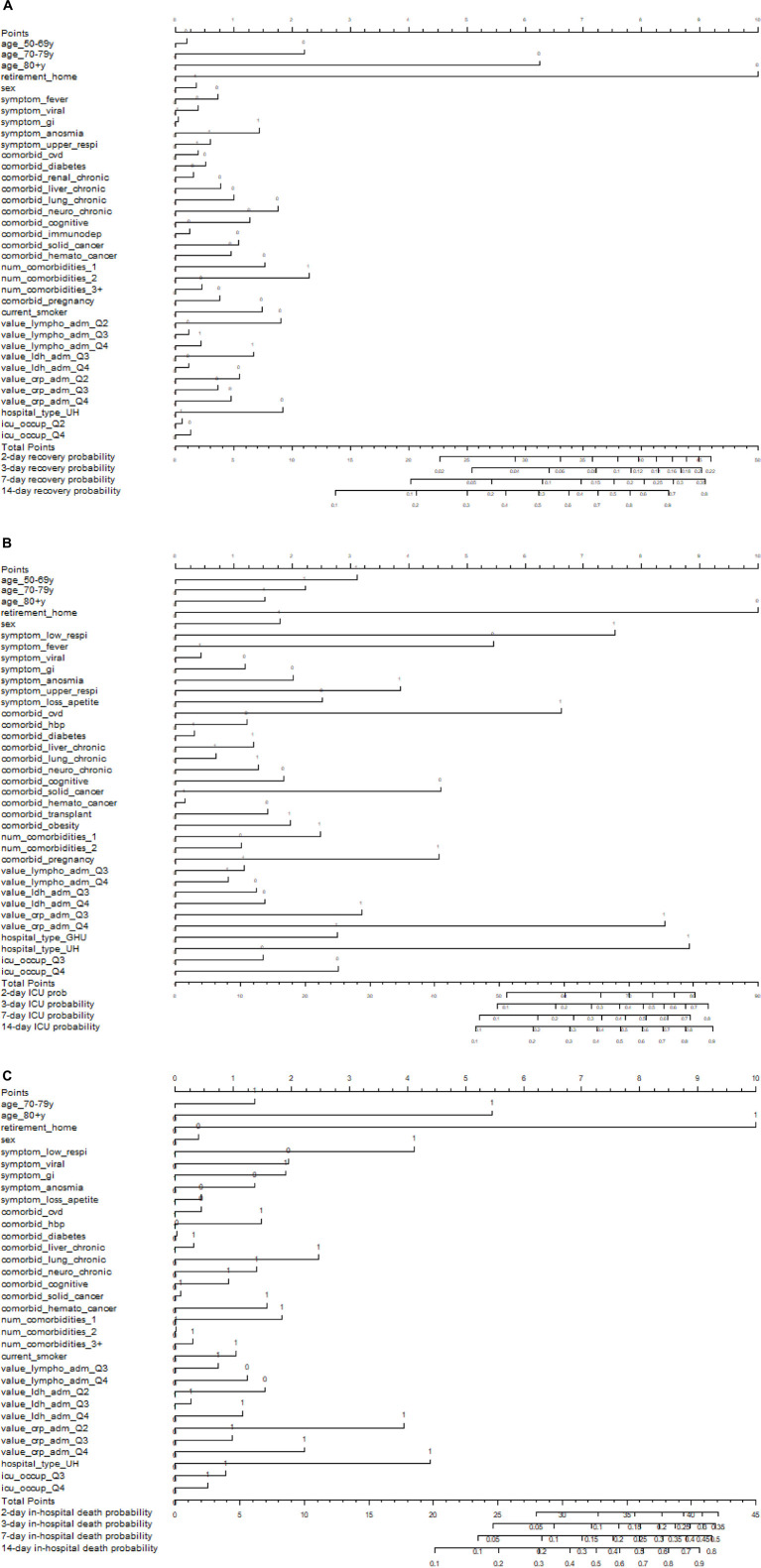
Nomograms to predict the transition probability for admission to recovery **(A)**, ICU **(B)**, and in-hospital death **(C)** after 2, 3, 7, and 14 days of hospitalisation in COVID-19 hospitalised COVID-19 patients. Patients’ values are located on the axis of each variable where 0 refers to “No” and 1 “Yes”; drawing an upward line at 90° angle to determine the number of points for that particular variable. The sum of these numbers of points is located on the total score axis; drawing a downward line at 90° angle to determine the probability of experiencing that particular transition at day 2, 3, 7, and 14.

Using the first three transitions in a competing risk framework, a nomogram was built assigning a score to each of the selected predictor variables associated with the recovery, ICU admission, and in-hospital death, respectively (Figure 4).

The transition from hospital admission to recovery was mainly predicted by age, receiving a number of 10 points when younger than 80+ plus 6.3 points when younger than 70–70 years and plus 2.2 points when younger than 50–69 years, and to a lesser extent by pregnancy (2.3 points) and the number of comorbidities (1.8 plus 1.5 plus 0.8 points when having less than 3 comorbidities, less than 2, and less than 1, respectively) and in particular the comorbidity of chronic neurological disorders (1.8 point when absent; Figure 4A). Points were lower than 1.5 for all other predictor variables relevant for recovery after hospital admission.

For a transition from hospital admission to ICU admission, the higher numbers of points were assigned to being of non-advanced age (10 when younger than 80+ plus 5.5 when not having a residence in a retirement home), high levels of CRP (8.8) and LDH (8.4) followed by hospital type (7.5 when admitted to university hospital), reporting the symptom of loss of appetite (6.6), and the lower points were for having not having cognitive disorders (4.5), but suffering from obesity (4.5) and reporting the symptom of lower respiratory complaints (3.9), and being male (3.1; Figure 4B). Points were lower than 3 for all other predictor variables relevant for ICU admission.

For the transition from hospital admission to in-hospital death, the higher numbers of points were assigned to advanced age (10 when 80+, 5.5 when 70–79 years, and 4.1 for residence in retirement home), followed by high levels of CRP (4.4) and LDH (3.9), and the comorbidity of chronic liver disease (2.5; Figure 4C). Points were lower than 2 for all other predictor variables relevant for in-hospital death after hospital admission.

For example, using the nomogram for calculating the transition probabilities for the first three transitions at 7 days after hospital admission for a reference patient resulted in a transition probability for ICU of 10%, for recovery of 50% and for in-hospital death less than 5%, while for the high-risk patient the transition probabilities of 40, 30, and 10%, respectively, corresponding with Figure 3B. Estimated c-index for the individual transitions, as illustrated by the nomograms, was 0.677 for the risk prediction of ICU transfer, and 0.629 and 0.766 for predicting recovery and in-hospital death, respectively, after hospital admission.

### Complete-case analysis

Patient’s characteristics of the complete-cases only did not differ from the 44,550 patients used for the MI (Supplementary Table 1). Less predictors were selected for each transition in the complete-case analysis (Supplementary Table 2), while showing a similar reduction in prediction error (data not shown). Predictors selected in both the imputed and complete-case dataset had regression coefficients pointing toward the same direction, and often of the same or a marginally weaker strength for the complete-case analysis.

## Discussion

In this study, a multi-state prediction model has been adopted for one of the first times to identify predictive factors of COVID-19 outcomes after hospitalisation, including recovery, ICU transfer (as proxy for critical disease state) and death, in a multi-state framework. Our findings indicate that all possible transitions are predicted by a different set of variables with varying magnitudes, but having in common the following variables: sex, age, levels of CRP and LDH, complaints related to the lower respiratory tract, and pre-existing comorbidities. The risk prediction model had good performance with 15% reduction in prediction error as compared to a null model derived from the Training set.

This study identified age, sex, symptoms of lower respiratory complaints, higher levels of CRP and LDH, as well as living in a retirement home, as the set of variables predicting progression to critical/fatal COVID-19, however varying in terms of hazard ratios for critical disease state and in-hospital death. These findings align with those of a meta-analysis using data of 69,762 patients, which reported age, cerebrovascular disease, CRP, LDH and cardiac troponin I as relevant prognostic factors for severe COVID-19 outcomes (36). In addition, an umbrella review and meta-analysis summarising estimates for 42 pre-existing comorbidities reported an increased risk of severe COVID-19 outcomes in patients with diabetes, obesity, cardiovascular disease, chronic obstructive pulmonary disease and dementia as well as liver cirrhosis and active cancer from studies conducted in Europe and US (37). In accordance with these results, our analysis found obesity to be a relevant predictor for ICU admission, and cardiovascular disease, chronic liver disease, neurological disorders and malignancies for in-hospital mortality, while recovery was predicted to be significant, but modestly lower for patients with one or more pre-existing conditions. Pre-existing comorbidities were also associated with higher mortality among COVID-19 patients previously admitted to ICU along with increasing age, high levels on disease severity scores, and disrupted immune-response (38). Also, in this study, increasing age and suffering from pre-existing conditions were selected to be of prognostic relevance for patients admitted to ICU with COVID-19. A note of caution is due here since results are based on a limited sample size of 6,520 ICU patients (15% of the total cohort).

Debate continues regarding the role of smoking status with COVID-19 severity. A recent meta-analysis including 517,020 patients from 109 studies found smoking to be significantly associated with ICU admission (OR: 1.73; 95% CI: 1.36, 2.19) and increased mortality (OR: 1.58, 95% CI: 1.38, 1.81) (39), in agreement with previous meta-analyses (40–42), while not confirmed by other meta-analyses (43–45) and recent large retrospective cohort studies (46, 47). This discrepancy could be attributed to the mediating role of comorbidities and their risk factors (46, 47) as well as the lower than expected prevalence of smoking among COVID-19 patients (44, 46, 47). The latter also validates the lower risk of SARS-CoV-2 infection observed among smokers (RR: 0.74; 95% CI: 0.58, 0.93), as established in a meta-analyses based on 45 studies providing data on SARS-CoV-2 infection in adults (45). The smoking rate of patients is, however, considered as insufficient to judge any association between smoking and COVID-19 because of the higher probability of smokers for being tested owing to symptoms similar to COVID-19 (48). In our study, smoking was not identified as a relevant strong predictor for COVID-19 severity (i.e., weak HRs only seen in the dataset with 10-fold MI), which concurs with the recorded lower smoking prevalence among included hospitalised COVID-19 patients (8.1%) as compared to the national prevalence (16.1%), as inquired by the Belgian Health Interview Survey of 2018 (49). However, with this apparently low proportion of smokers requiring hospitalisation for their COVID-19, caution must be applied, as an “unknown” smoking status was recorded for around 40% of the patients considered for inclusion, without any reason provided but presumably more likely among smokers. In general, it appears that, if existing, any predictive value of current smoking status on critical/fatal COVID-19 outcomes is likely to be small, but this needs to clarified with more accurate data.

Considering the choice of the methodology for the predictive models, a systematic review, including 107 risk prediction models for severe COVID-19 outcomes, concluded that many of the proposed models carry a high risk of bias because of inappropriate statistical methods ignoring time-to-event and the presence of competing risks (2). Integrating competing risk models into a multi-state framework allows simultaneous modelling of time-to-event outcomes and disease progression, and hereby enables the calculation of transition and state occupation probabilities, adding an extra layer of information (18, 50). Like this, the multi-state model more accurately describes the evolution of hospitalised patients, by accounting for intermediate events of disease progression that likely influence disease outcomes over time, i.e., time and event-related dependencies of disease progression. Similar to a smaller sample size study covering only one region of Spain (51), the present study devised the multi-state framework for building a risk prediction model for COVID-19 critical state (ICU transfer) and death versus recovery, and consistently co-morbidities were more frequently observed to be the more prevalent among those experiencing worst COVID-19 outcomes, like hospitalisation, and subsequently in-hospital death. In addition, we thereby identified male sex, high levels of CRP and LDH as well as reporting lower respiratory complaints as common important predictive factors for transitioning to ICU or in-hospital death after hospitalisation due to SARS-CoV-2 infection, with additionally middle-age, obesity, reporting loss of appetite and staying in a university hospital for a transfer to ICU, while advanced age and a higher number of comorbidities for in-hospital death.

The use of the nationwide hospital surveillance data, along with appropriate statistical models fitting the hospital setting, are of crucial importance for informed evidence-based clinical decision-making. In the present study, the underlying time-to-event analyses allowed prediction of the state-occupation probabilities through the transition hazards and cumulative hazards, i.e., an indirect approach by which variable selection for the prediction model occurred at the level of transition hazards of the time-to-event models instead of state-occupation probabilities. Alternatively, a pseudo-value regression model would offer the possibility to directly model the state-occupation probabilities for intermediate and absorbing state, and modelling this only for a predefined future time t after hospital admission, as chosen in advance (28, 29). Further, information on when the event occurred, as needed to account for time dependencies, was in our data only reported for hospital admission and discharge (i.e., in-hospital death vs. recovery) and for admission to ICU, and therefore this latter was the only intermediate event considered as a proxy for critical disease progression. Also, the lack of information on withdrawal and/or the Limitation of Life-Sustaining Therapies might limit overall conclusions. However, with the large number of hospitalised COVID-19 patients in Belgium, it is anticipated to have a sufficient number of events per variable to, despite the presence of low-prevalence predictors, eliminate bias in the regression coefficients and improve predictive accuracy for all five transitions considered (52, 53). Albeit the large sample size of our study allowed us to use a random split approach for internal validation, other internal validation tests, such as bootstrapping or cross-validation, should be used to fully account for overfitting and optimism in model performance (54). The Brier score, used for the overall evaluation of the model performance as well as the individual transitions, limited comparison with other established published prediction models, though this evaluation supported by providing the c-index for the individual transitions as illustrated in the nomogram. This prediction model is based on patient’s electronic medical files relying on the clinicians’ report of clinical observations and anamnesis and medical registration personnel reporting accurately, which might vary across hospitals and during the peak periods of the pandemic. In this respect, an important limitation is the number of missing values without any reason for the incomplete data, in particular an issue for current smoking. A complete-case analysis was performed to confirm the results obtained after MI, but warrants caution because of the underlying assumption of missing at random. Finally, the model’s flexibility for adaptation to other settings and countries remains to be examined, by way of an external validation judging not only discrimination but also calibration and clinical utility in a dataset with a minimum of 100 events collected using appropriate study designs and representative of the target population (55).

In conclusion, integrating the standard Cox models into a multi-state framework allows the study of separate competing outcomes simultaneously as well as the disease progression through intermediate states. This paper shows the application of the multi-state framework for the deployment of a risk prediction model for COVID-19 disease progression after hospitalisation. Each transition, i.e., from hospitalisation to critical disease state, and subsequently to recovery or in-hospital death, has its own set of variables with varying magnitudes, and commonly including sex, age, levels of CRP and LDH, complaints related to the lower respiratory tract, and pre-existing comorbidities as predicting variables. The deployment in a risk score to predict the first three potential transitions shows the potential to be utilised by potential stakeholders, such as health care providers and policy makers, for informed clinical decision on patients’ management, and resource allocation for effective health systems.

## Data availability statement

The data that support the findings of this study are available from Sciensano, but restrictions apply to the availability of these data, which were used under licence for the current study, and so are not publicly available. Data are however available from the corresponding author upon reasonable request and with permission of Sciensano.

## Membership of the Belgian collaborative group on COVID-19 hospital surveillance

The Belgian Collaborative Group on COVID-19 Hospital Surveillance consists of all local investigators who are responsible for the design of the COVID surveillance, identifying variables for inclusion, and data collection in each of the hospitals. They have all approved the final manuscript. Anke Vanhoenacker, Ziekenhuisnetwerk Antwerpen, Belgium; Benjamin Kerzmann, Clinique Notre Dame de Grâce, Gosselies, Belgium; BS, Sciensano, Brussels, Belgium; Camelia Rossi, Centre Hospitalier Universitaire Ambroise Paré, Mons, Belgium; Carole Schirvel, CHIREC, Brussels, Belgium; Caroline Gheysen, Jan Yperman Ziekenhuis, Poperinge, Belgium; Catherine Nachtergal, Cliniques de l’Europe, Brussels, Belgium; Chloé Wyndham-Thomas, Sciensano, Brussels, Belgium.; Denis Piérard, Universitair Ziekenhuis Brussel, Brussels; Didier Delmarcelle, Clinique St. Jean, Brussels, Belgium; Dominique Van Beckhoven, Sciensano, Brussels, Belgium; Elise Willems, Algemeen Ziekenhuis Nikolaas, Sint-Niklaas, Belgium; Erica Sermijn, A.S.Z. Ziekenhuis, Aalst, Belgium; Eva Van Braeckel, Universiteit Gent, Gent, Belgium; Fabio Taccone, Hôpital Erasme, Brussels, Belgium; Filip Triest, Algemeen Ziekenhuis Sint Lucas, Gent, Belgium; Frank Staelens, Onze Lieve Vrouwziekenhuis, Aalst, Belgium; Geert Meyfroidt, Katholieke Universiteit Leuven, Leuven, Belgium; Jean-Marc Minon, Centre Hospitalier Régional de la Citadelle, Liège, Belgium; Jenny Chung, Sciensano, Brussels, Belgium; Jens Van Praet, Algemeen Ziekenhuis Sint Jan, Brugge-Oostende, Belgium; Koen Blot, Sciensano, Brussels, Belgium; Kristof Bafort, Mariaziekenhuis, Pelt, Belgium; Leïla Belkhir, Cliniques Universitaires Saint-Luc, Brussels, Belgium; Mélanie Delvallee, Centre Hospitalier de Wallonie Picarde, Tournai, Belgium; Nathalie Bossuyt, Sciensano, Brussels, Belgium; Nicolas Dauby, Centre Hospitalier Universitaire Saint-Pierre, Brussels, Belgium; Nina Van Goethem, Sciensano, Brussels, Belgium; Paul De Munter, Universitair Ziekenhuis Leuven, Leuven, Belgium; Philippe Minette, Centres Hospitaliers Jolimont, Belgium; Pierre Yves Machurot, Centre Hospitalier de l’Ardenne, Belgium; Rémy Demeester, Centre Hospitalier Universitaire de Charleroi, Charleroi, Belgium; Robby De Pauw, Sciensano, Brussels, Belgium; Roeland Verstraete, Algemeen Ziekenhuis, Monica, Antwerpen, Belgium; Samy Amir Aouachria, Centre Hospitalier Chrétien, Liège, Belgium; Saphia Mokrane, Hôpitaux Iris Sud, Brussels, Belgium; Sarah Cooreman, Algmeen Ziekenhuis Monica, Antwerp, Belgium; Sarah Loof, Algemeen Ziekenhuis Maria Middelares Gent, Gent, Belgium; Séverine Noirhomme, Centre Hospitalier Régional de Namur, Belgium; Steven Callens, Universiteit Gent, Gent, Belgium; Thierry Dugernier, Clinique Saint-Pierre, Ottignies, Belgium; Vincent Colombie, Centre Hospitalier Epicura, Baudour, Belgium; Xavier Holemans, Grand Hôpital de Charleroi, Charleroi, Belgium.

## Author contributions

EM and JP: concept and design, statistical analysis, and interpretation of data. BS and MV: provision of data, curation of data, and acquisition of data. EM: drafting of the manuscript. EM, BS, MV, and JP: critical revision of the manuscript for important intellectual content. JP: supervision. All authors contributed to the article and approved the submitted version.
