# Supplementary material for: Predicting COVID-19 progression in hospitalized patients in Belgium from a multi-state model
Source: Front Med (Lausanne). 2022 Nov 23;9:1027674. doi: 10.3389/fmed.2022.1027674 (PMC9727386; doi:10.3389/fmed.2022.1027674)
Supplement: Supplementary file 1 [file Table_1.pdf]

# *Supplementary Material*

## Supplementary Tables

**Supplementary Table 1** Description of the information of COVID-19 patients (complete-cases only) according to health states during hospitalisation available from the Belgian COVID-19 hospital surveillance database between March 2020 and June 2021.

| | | | Final health states | | |
|---|---|---|---|---|---|
| | Hospitalisation (N=18,994) | Admission to ICU (N=2,749) | Recovery (N=15,547) | In-hospital death (N=2,926) | Lost to follow-up (N=521) |
| Age (years) | | | | | |
| 18-49 | 3,246 (17.1%) | 441 (16.0%) | 3,108 (20.0%) | 53 (1.8%) | 85 (16.3%) |
| 50-69 | 6,512 (34.3%) | 1,295 (47.1%) | 5,814 (37.4%) | 481 (16.4%) | 217 (41.7%) |
| 70-79 | 4,000 (21.1%) | 735 (26.7%) | 3,141 (20.2%) | 731 (25.0%) | 128 (24.6%) |
| 80+ | 5,236 (27.6%) | 278 (10.1%) | 3,484 (22.4%) | 1,661 (56.8%) | 91 (17.5%) |
| Median [IQR] | 69 [55, 81] | 65 [55, 73] | 66 [53, 78] | 81 [73, 87] | 67 [57, 76] |
| Residence in retirement home | 2,165 (11.4%) | 107 (3.9%) | 1,401 (9.0%) | 746 (25.5%) | 18 (3.5%) |
| Sex (males) | 10,509 (55.3%) | 1,856 (67.5%) | 8,477 (54.5%) | 1,727 (59.0%) | 305 (58.5%) |
| Symptoms at admission | | | | | |
| Lower respiratory | 13,667 (72.0%) | 2,324 (84.5%) | 11,076 (71.2%) | 2,205 (75.4%) | 386 (74.1%) |
| Fever | 9,422 (49.6%) | 1,597 (58.1%) | 7,780 (50.0%) | 1,368 (46.8%) | 274 (52.6%) |
| Typical viral infection | 8,658 (45.6%) | 1,307 (47.5%) | 7,445 (47.9%) | 1,004 (34.3%) | 209 (40.1%) |
| Gastro-intestinal | 5,341 (28.1%) | 678 (24.7%) | 4,658 (30.0%) | 562 (19.2%) | 121 (23.2%- |
| Anosmia | 1,657 (8.7%) | 239 (8.7%) | 1,496 (9.6%) | 112 (3.8%) | 49 (9.4%) |
| Upper respiratory | 1,505 (7.9%) | 223 (8.1%) | 1,303 (8.4%) | 156 (5.3%) | 46 (8.8%) |
| Loss of appetite | 388 (2.0%) | 173 (6.3%) | 336 (2.2%) | 51 (1.7%) | 1 (0.2%) |
| Pre-existing conditions | | | | | |
| Cardiovascular disease | 6,094 (32.1%) | 890 (32.4%) | 4,380 (28.2%) | 1,562 (53.4%) | 152 (29.2%) |
| Arterial hypertension | 7,944 (41.8%) | 1,247 (45.4%) | 6,073 (39.1%) | 1,631 (55.7%) | 240 (46.1%) |
| Diabetes mellitus | 4,588 (24.2%) | 767 (27.9%) | 3,526 (22.7%) | 913 (31.2%) | 149 (28.6%) |
| Chronic renal disease | 2,577 (13.6%) | 319 (11.6%) | 1,827 (11.8%) | 686 (23.4%) | 64 (12.3%) |
| Chronic liver disease | 503 (2.6%) | 98 (3.6%) | 384 (2.5%) | 107 (3.7%) | 12 (2.3%) |
| Chronic lung disease | 3,128 (16.5%) | 553 (20.1%) | 2,362 (15.2%) | 657 (22.5%) | 109 (20.9%) |
| Neurological disorders | 1,493 (7.9%) | 151 (5.5%) | 1,084 (7.0%) | 382 (13.1%) | 27 (5.2%) |
| Cognitive disorders | 1,818 (9.6%) | 98 (3.6%) | 1,247 (8.0%) | 542 (18.5%) | 29 (5.6%) |
| Immunosuppressive condition | 390 (2.1%) | 105 (3.8%) | 300 (1.9%) | 80 (2.7%) | 10 (1.9%) |
| Cancer | | | | | |
| Solid | 1,824 (9.6%) | 222 (8.1%) | 1,344 (8.6%) | 428 (14.6%) | 52 (10.0%) |
| Haematological | 401 (2.1%) | 88 (3.2%) | 263 (1.7%) | 131 (4.5%) | 7 (1.3%) |
| Transplant | 118 (0.6%) | 40 (1.5%) | 90 (0.6%) | 23 (0.8%) | 5 (1.0%) |
| Obesity | 3,020 (15.9%) | 714 (26.0%) | 2,485 (16.0%) | 417 (14.3%) | 118 (22.6%) |
| Number of comorbidities | | | | | |
| 0 | 4,720 (24.9%) | 583 (21.2%) | 4,413 (28.4%) | 192 (6.6%) | 115 (22.1%) |
| 1 | 4,437 (23.4%) | 636 (23.1%) | 3,789 (24.4%) | 514 (17.6%) | 134 (25.7%) |
| 2 | 4,179 (22.0%) | 633 (23.0%) | 3,310 (21.3%) | 760 (26.0%) | 109 (20.9%) |
| 3+ | 5,658 (29.8%) | 897 (32.6%) | 4,035 (26.0%) | 1,460 (49.9%) | 163 (31.3%) |

| | | | | | |
|---|---|---|---|---|---|
| Risk factors | | | | | |
| Pregnancy | 129 (0.7%) | 16 (0.6%) | 127 (0.8%) | 0 (0%) | 2 (0.4%) |
| Current smoker | 1,544 (8.1%) | 242 (8.8%) | 1,273 (8.2%) | 208 (7.1%) | 63 (12.1%) |
| Laboratory parameters at admission (Median [IQR]) | | | | | |
| Lymphocytes (n/mm³) | 830 [460, 1270] | 750 [410,1150] | 870 [500, 1310] | 640 [330,1040] | 660 [139,1052] |
| LDH (IU/L) | 332 [252, 451] | 419 [313,574] | 321 [246, 431] | 396 [293,558] | 376 [276,525] |
| CRP (mg/dL) | 64 [26, 123] | 109 [52, 179] | 59 [23, 113] | 95 [46, 164] | 89 [35, 152] |
| Hospital characteristics | | | | | |
| Hospital type | | | | | |
| GH | 12,909 (68.0%) | 1,555 (56.6%) | 10,525 (67.7%) | 1,995 (68.2%) | 389 (74.7%) |
| GHU | 4,499 (23.7%) | 680 (24.7%) | 3,650 (23.5%) | 735 (25.1%) | 114 (21.9%) |
| UH | 1,586 (8.4%) | 514 (18.7%) | 1,372 (8.8%) | 196 (6.7%) | 18 (3.5%) |
| ICU occupancy (%, Median [IQR])[a] | 0.42 [0.25,0.64] | 0.40 [0.24,0.59] | 0.42 [0.25,0.63] | 0.44 [0.25,0.67] | 0.50 [0.25,0.67] |
| Clinical features | | | | | |
| Hospital length of stay (days, Median [IQR]) | 9 [5, 14] | 17 [10, 28] | 9 [5, 14] | 10 [6, 16] | 8 [4, 16] |
| Time from hospital admission to ICU admission (days, Median [IQR]) | - | 1 [0, 3] | - | - | - |
| ICU transfer | 2,749 (14.5%) | 2,749 (100%) | 1,672 (10.8%) | 870 (29.7%) | 207 (39.7%) |
| Final health status | | | | | |
| Discharged alive | 15,547 (81.9%) | 1,672 (60.8%) | 15,547 (100%) | 0 (0%) | 0 (0%) |
| In-hospital death | 2,926 (15.4%) | 870 (31.6%) | 0 (0%) | 2,926 (100%) | 0 (0%) |
| Lost to follow-up | 521 (2.7%) | 207 (7.5%) | 0 (0%) | 0 (0%) | 521 (100%) |

Values are numbers and percentage, or median and interquartile values

Abbreviations: CRP, c-reactive protein; GH, general hospital; GHU, General Hospital with University characteristics; ICU, intensive care units; IQR, interquartile range; LDH, lactate dehydrogenase; UH, university hospital

[a] ICU occupancy taken at admission for hospitalised patients without a transfer to ICU, and for ICU patients taken at the time of their ICU transfer instead.

**Supplementary Table 2** Hazard ratios and 95% confidence intervals for transitions of COVID-19 disease progression in a multi-state risk prediction setting in a complete-case analysis.

| Predictors | **Transition 1 Hospitalisation --> ICU** | **Transition 2 Hospitalisation --> Recovery** | **Transition 3 Hospitalisation --> in-hospital death** | **Transition 4 ICU --> Recovery** | **Transition 5 ICU --> In-hospital death** |
|---|---|---|---|---|---|
| Age (years) | | | | | |
| Q2 vs Q1 | 1.23 (1.12; 1.35) | 0.83 (0.78; 0.88) | | | |
| Q3 vs Q1 | | 0.53 (0.50; 0.57) | 1.80 (1.49; 2.17) | 0.75 (0.66; 0.85) | 1.12 (0.94; 1.33) |
| Q4 vs Q1 | 0.42 (0.36; 0.50) | 0.37 (0.34; 0.39) | 4.05 (3.38; 4.85) | | 1.65 (1.22; 2.24) |
| Sex (males) | 1.28 (1.16; 1.41) | | 1.22 (1.10; 1.36) | | |
| Residence in retirement home | 0.75 (0.60; 0.92) | | 1.62 (1.43; 1.83) | | 1.02 (0.66; 1.56) |
| Symptoms at admission | | | | | |
| Lower respiratory | 1.33 (1.18; 1.51) | | 1.40 (1.24; 1.57) | | |
| Fever | 1.03 (0.94; 1.13) | | | | |
| Typical viral infection | | 1.07 (1.03; 1.12) | 0.91 (0.81; 1.02) | | |
| Gastro-intestinal | 0.86 (0.78; 0.96) | 1.05 (1.00; 1.09) | 0.87 (0.76; 0.99) | | |
| Anosmia | | 1.08 (1.00; 1.16) | 0.82 (0.62; 1.09) | | |
| Upper respiratory | | | | | |
| Loss of appetite | 1.74 (1.38; 2.19) | | | 1.19 (0.93; 1.53) | |
| Pre-existing conditions | | | | | |
| Cardiovascular disease | | 0.97 (0.91; 1.02) | 1.20 (1.05; 1.36) | | 1.23 (1.01; 1.50) |
| Arterial hypertension | 1.01 (0.92; 1.11) | | | | |
| Diabetes mellitus | | 0.95 (0.89; 1.00) | 1.03 (0.90; 1.17) | 0.95 (0.82; 1.10) | |
| Chronic renal disease | | 0.91 (0.85; 0.97) | | | |
| Chronic liver disease | | 0.99 (0.88; 1.13) | 1.15 (0.84; 1.58) | | |
| Chronic lung disease | 1.07 (0.96; 1.21) | 0.92 (0.87; 0.98) | | 0.94 (0.81; 1.10) | |
| Neurological disorders | 0.98 (0.82; 1.17) | 0.82 (0.76; 0.89) | 1.28 (1.10; 1.50) | | |
| Cognitive disorders | 0.87 (0.70; 1.07) | 0.87 (0.81; 0.94) | 1.05 (0.92; 1.21) | | |
| Immunosuppressive conditions | | 0.96 (0.83; 1.11) | | | |
| Cancer | | | | | |
| Solid | | 0.96 (0.89; 1.03) | 1.25 (1.07; 1.46) | | |

| | | | | | |
|---|---|---|---|---|---|
| Haematological | | 0.88 (0.77; 1.02) | 1.19 (0.88; 1.62) | | |
| Transplant | 1.08 (0.70; 1.67) | | | | |
| Pregnancy | | 1.17 (0.89; 1.53) | | | |
| Obesity | 1.39 (1.25; 1.55) | | 0.94 (0.78; 1.12) | | |
| Number of comorbidities | | | | | |
| 1 vs 0 | | | | | |
| 2 vs 0 | | 0.88 (0.83; 0.93) | 1.03 (0.87; 1.21) | | |
| 3+ vs 0 | | 0.83 (0.76; 0.90) | 1.22 (1.01; 1.48) | 0.96 (0.82; 1.11) | 1.07 (0.89; 1.30) |
| Risk factors | | | | | |
| Current smoker | | | | | |
| Laboratory parameters at admission | | | | | |
| Lymphocytes (n/mm$^3$) | | | | | |
| Q2 vs Q1 | | | | | |
| Q3 vs Q1 | | 1.03 (0.98; 1.08) | 0.87 (0.76; 0.99) | | |
| Q4 vs Q1 | | 1.14 (1.09; 1.20) | 0.81 (0.71; 0.94) | | |
| LDH (IU/L) | | | | | |
| Q2 vs Q1 | | | | | |
| Q3 vs Q1 | 1.16 (1.03; 1.31) | 0.96 (0.91; 1.00) | 1.10 (0.96; 1.26) | | |
| Q4 vs Q1 | 1.93 (1.73; 2.15) | 0.88 (0.84; 0.93) | 1.84 (1.62; 2.09) | 0.98 (0.87; 1.11) | |
| CRP (mg/dL) | | | | | |
| Q2 vs Q1 | | | 1.01 (0.85; 1.18) | | |
| Q3 vs Q1 | 1.05 (0.93; 1.19) | 0.98 (0.93; 1.03) | 1.13 (0.96; 1.32) | | |
| Q4 vs Q1 | 1.91 (1.71; 2.13) | 0.89 (0.84; 0.94) | 1.48 (1.26; 1.73) | 0.93 (0.82; 1.05) | |
| Hospital characteristics | | | | | |
| Hospital type at admission | | | | | |
| GHU vs GH | | | | | |
| UH vs GH | 2.04 (1.77; 2.36) | 1.02 (0.94; 1.11) | 0.95 (0.75; 1.20) | | |
| ICU occupancy [a] | | | | | |
| Q2 vs Q1 | | 1.02 (0.97; 1.07) | | | |
| Q3 vs Q1 | | | 1.08 (0.95; 1.24) | | |
| Q4 vs Q1 | 0.89 (0.80; 0.99) | | 1.12 (0.99; 1.27) | | |

Abbreviations: CRP, c-reactive protein; GH, general hospital; GHU, General Hospital with University characteristics; ICU, intensive care units; LDH, lactate dehydrogenase; UH, university hospital; Q, quartile;
[a] ICU occupancy taken at admission for hospitalised patients without a transfer to ICU, and for ICU patients taken at the time of their ICU transfer instead.
